# Yield of Rare Variants Detected by Targeted Next-Generation Sequencing in a Cohort of Romanian Index Patients with Hypertrophic Cardiomyopathy

**DOI:** 10.3390/diagnostics10121061

**Published:** 2020-12-07

**Authors:** Miruna Mihaela Micheu, Nicoleta-Monica Popa-Fotea, Nicoleta Oprescu, Stefan Bogdan, Monica Dan, Alexandru Deaconu, Lucian Dorobantu, Oana Gheorghe-Fronea, Maria Greavu, Corneliu Iorgulescu, Alexandru Scafa-Udriste, Razvan Ticulescu, Radu Gabriel Vatasescu, Maria Dorobanțu

**Affiliations:** 1 Department of Cardiology, Clinical Emergency Hospital of Bucharest, Calea Floreasca 8, 014461 Bucharest, Romania; nicoleta_m_oprescu@yahoo.com (N.O.); stefan_n_bogdan@yahoo.com (S.B.); mnc_dan@yahoo.com (M.D.); alexandru_deaconu@yahoo.com (A.D.); ludorobantu@gmail.com (L.D.); clarresearch@yahoo.com (O.G.-F.); iorgulescu_corneliu@yahoo.com (C.I.); alexscafa@yahoo.com (A.S.-U.); radu_vatasescu@yahoo.com (R.G.V.); maria.dorobantu@gmail.com (M.D.); 2 Department 4-Cardiothoracic Pathology, University of Medicine and Pharmacy Carol Davila, Eroii Sanitari Bvd. 8, 050474 Bucharest, Romania; 3 Monza Hospital, Tony Bulandra Street, No. 27, 021967 Bucharest, Romania; r0zmaria@yahoo.com (M.G.); razvanticulescu@gmail.com (R.T.)

**Keywords:** hypertrophic cardiomyopathy, next-generation sequencing, rare genetic variants

## Abstract

Background: The aim of this study was to explore the rare variants in a cohort of Romanian index cases with hypertrophic cardiomyopathy (HCM). Methods: Forty-five unrelated probands with HCM were screened by targeted next generation sequencing (NGS) of 47 core and emerging genes connected with HCM. Results: We identified 95 variants with allele frequency < 0.1% in population databases. MYBPC3 and TTN had the largest number of rare variants (17 variants each). A definite genetic etiology was found in 6 probands (13.3%), while inconclusive results due to either known or novel variants were established in 31 cases (68.9%). All disease-causing variants were detected in sarcomeric genes (MYBPC3 and MYH7 with two cases each, and one case in TNNI3 and TPM1 respectively). Multiple variants were detected in 27 subjects (60%), but no proband carried more than one causal variant. Of note, almost half of the rare variants were novel. Conclusions: Herein we reported for the first time the rare variants identified in core and putative genes associated with HCM in a cohort of Romanian unrelated adult patients. The clinical significance of most detected variants is yet to be established, additional studies based on segregation analysis being required for definite classification.

## 1. Introduction

Hypertrophic cardiomyopathy (HCM) is the most common inherited cardiac illness, affecting at least 1 in 500 individuals in the general population [1,2]. It is defined by the presence of left ventricular hypertrophy (LVH) not solely explained by abnormal loading conditions [3].

Due to numerous genetic and non-genetic modifiers yet to be deciphered, clinical expression and outcomes are particularly diverse, varying from asymptomatic to severe forms or even sudden cardiac death [4]. The genetic basis is complex, mainly involving variation in sarcomeric genes, but mutation in other genes can cause mimicking pathologies with isolated HCM or with complex phenotypes comprising LVH [5]. The main causative genes are cardiac myosin binding protein C (*MYBPC3*) and β-myosin heavy chain (*MYH7*); together they are accountable for approximatively half of all HCM cases and for at least 75% of genotype-positive probands [6]. Amongst 57 candidate genes recently curated, these 2 genes along with other 6 (listed in bold letters in Table 1) have been designated as having definitive evidence for HCM and therefore should be part of clinical genetic testing [7,8].

Increased use of high-throughput sequencing techniques together with comprehensive gene panels led to detection of novel disease-causing variants, but mainly increased the detection of variants of uncertain significance (VUS) which are difficult to interpret, particularly in case of “private” mutations unique to a single family.

Notably, the underlying etiology may vary across different populations, precisely the probability of obtaining a positive result is influenced by the existence of preceding studies in the respective population [9]. Compared to large statistics concerning the spectrum of HCM variants in Western and Northern Europe [10,11,12,13], information about the genetic basis of HCM in Romanian adult population is limited; hence, we aimed to investigate the HCM-related rare variants in a cohort of Romanian index cases.

## 2. Materials and Methods

### 2.1. Study Population

The study was approved by the Ethics Committee of the Clinical Emergency Hospital of Bucharest, and performed in compliance with the principles of the Declaration of Helsinki. Before enrolment, written informed consent was obtained from all subjects. The study population comprised 45 unrelated HCM probands referred to our center for standard medical care and/or genetic testing between 2017 and 2020. HCM was diagnosed according to criteria issued by European Society of Cardiology (ESC), namely increased left ventricular (LV) wall thickness (≥15 mm in adults) not solely explained by abnormal loading conditions [5]. All patients underwent comprehensive clinical work-up, including personal and family medical history, physical examination, 12-lead electrocardiogram, two-dimensional transthoracic echocardiography, and genetic testing.

### 2.2. Genetic Testing

The genetic testing methodology has been previously reported [14]. Briefly, blood samples were collected at enrolment and total DNA was isolated using MagCore Genomic DNA Whole Blood Kit (RBC Bioscience) following the manufacturer’s protocol, and subsequently being quantified using Qubit dsDNA HS assay kit (Life Technologies). Targeted next generation sequencing (NGS) was performed on an Illumina MiSeq platform using TruSight Cardio Sequencing Kit (Illumina) according to manufacturer’s instructions. An initial amount of 50 ng of genomic DNA was used for optimal gene enrichment.

### 2.3. Variant Assessment

Data files yielded during sequencing runs were processed by MiSeq Reporter software (Illumina) to generate FASTQ files, and to perform the mapping of reads against the reference human genome (GRCh37) using Burrows–Wheeler Aligner-Maximal Exact Match (BWA-MEM) algorithm [15]. Following alignment, variant calling was done with Genome Analysis Toolkit (GATK) and Variant Call Format (VCF) files were produced as output. VCF files were analyzed with VariantStudio v3.0 software (Illumina).

The following filters were used to select the candidate variants for further analysis: include list of 47 genes associated with HCM (Table 1), protein-coding variants, high quality calling (PASS filter), allele frequency (AF) < 0.1% in population databases. The cut-off of 0.1% was chosen considering the disease prevalence in general population (1 in 500 individuals or 1/1000 chromosomes) [1].

Sequence variants passing the aforesaid filters were analyzed individually and were further reported using Human Genome Variation Society standardized nomenclature [16]. Interpretation of clinical significance followed the joint consensus recommendations of American College of Medical Genetics and Genomics and the Association for Molecular Pathology (ACMG/AMP), taking into account evidences such as allele frequency in control populations and predicted effect on the resultant protein [17]. Variant frequency was determined using the allele frequency estimates from the 1000 genomes project (GRCh37 reference assembly) and gnomAD (v2.1.1 dataset aligned against the GRCh37 reference) (accessed on August 2020); AF was retrieved from total population frequencies, including controls within gnomAD v2.1.1. For prediction of functional consequence of missense variants four freely available online in silico tools were used: Sorting Tolerant from Intolerant (SIFT), Protein variation effect analyzer (Provean), PolyPhen-2, and Mutation Taster. The disease-causing potential of stop-gain and stop-loss variants, splicing variants, frameshift, and in-frame insertions and deletions was estimated with Mutation Taster. Accordingly, a five-tier system was used to classify the variants into one of the categories: benign (B), likely benign (LB), variant of uncertain significance (VUS), likely pathogenic (LP), or pathogenic (P).

Each variant was subsequently cross-referenced with its classification provided by publicly accessible databases: the NCBI ClinVar database and the Human Gene Mutation Database (HGMD) (accessed on August 2020). In addition, all novel detected variants (irrespective of in silico prediction) were examined using VarSome [18]—a human genomic variant search engine (accessed on November 2020), and classified accordingly.

### 2.4. Variant Databases and In Silico Tools

We accessed the following variant databases: 1000 Genomes Project (https://www.internationalgenome.org/1000-genomes-browsers), the Exome Variant Server from the NHLBI Exome Sequencing Project (ESP) (https://esp.gs.washington.edu/EVS/), NCBI dbSNP (http://www.ncbi.nlm.nih.gov/SNP/), Genome Aggregation Database (gnomAD; http://gnomad.broadinstitute.org), ClinVar (https://www.ncbi.nlm.nih.gov/clinvar/), Human Genome Mutation Database (5-day trial license HGMD Professional 2020.2; http://www.biobase-international.com/), VarSome (https://varsome.com/).

In silico tools used in this study were as it follows: SIFT (https://sift.bii.a-star.edu.sg/), PolyPhen-2 (http://genetics.bwh.harvard.edu/pph2/), Provean (http://provean.jcvi.org), and MutationTaster (http://www.mutationtaster.org/).

### 2.5. Statistical Analysis

Data were analyzed using SPSS Statistics (version 23.0); results were presented as mean ± standard deviation for continuous variables and n (%) for categorical variables.

## 3. Results

### 3.1. Study Population

Forty-five unrelated index patients (33 men and 12 women) with HCM were studied. The mean age at enrolment was 51 years (SD 15.5, range 21 to 87 years). When dividing the HCM cohort into positive, considering those with a definite genetic etiology, and negative, those without definitive genetic results, the mean age in the positive group was significantly lower, 34 ± 10.3 years (range 21 to 48), compared with the negative one, 53 ± 14.7 years (range 25 to 87), *p* = 0.04. Except of the age difference between the two group, no other statistically significant differences were found in the clinical presentation or general characteristics of HCM cohort. Maximal LV wall thickness was 20.8 ± 5.2 mm (range 15 to 38 mm) in the overall cohort, with no differences between those with or without definitive genetic diagnosis, and moreover, no differences were found in various echocardiographic parameters (Table 2).

### 3.2. Genes and Variants

Of the 174 genes covered by TruSight Cardio Sequencing Kit, only 47 genes were considered in this analysis, including the 8 core sarcomeric genes robustly associated with HCM (*ACTC1*, *MYBPC3*, *MYH7*, *MYL2*, *MYL3*, *TNNI3*, *TNNT2*, *TPM1*). Additionally, non-sarcomeric genes reported to be connected with isolated HCM or with complex phenotypes comprising LVH, were studied. The complete list of analyzed genes is depicted in Table 1.

**Figure 1:**
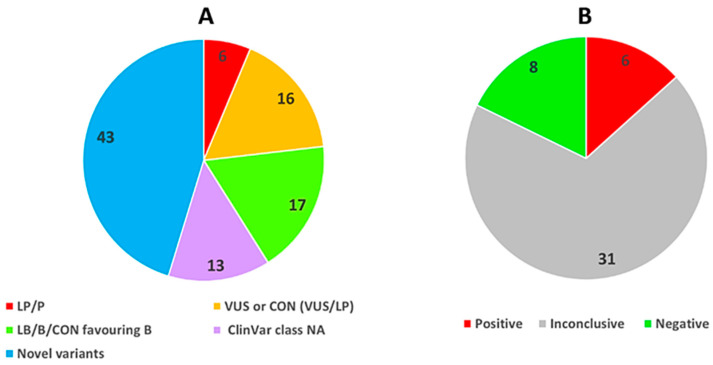
Percentage distribution of rare variants (AF < 0.001) and detection rates. (**A**). Type and distribution of variants according to ClinVar classification; novel variants refers to sequence variants not previously published nor reported in online variant databases; mutations within all the others groups were previously published or reported in specific databases. (**B**). Results of genetic testing within the entire HCM cohort broken down by category. Positive: all cases with a variant classified as LP/P by ClinVar; negative: no rare variant identified or only B/LB variants according to ClinVar; inconclusive: cases with one (or combination) of the following type of variants: variants categorized as VUS, variants for which conflicting interpretations of pathogenicity exists (either VUS/LP or VUS/B/LB), variants without ClinVar classification, and all novel variants irrespective of VarSome classification. AF allele frequency; B benign; CON variant with conflicting interpretations of pathogenicity; LB likely benign; LP likely pathogenic; NA data not available; P pathogenic; VUS variant of uncertain significance.

After filtering, a total of 95 distinct rare variants in 33 genes were found in 37 of 45 probands, providing an average of 2 variants per index case (Figure 1 and Table 3, Table 4 and Table 5). All variants were identified in heterozygosis. The mean depth of sequence coverage across target regions was 202x (ranged from 25 to 741). The *MYBPC3* and *TTN* genes had the largest number of rare variants (17 variants each), followed by *MYH7* (9 variants). Altogether, there were 65 missense variants (68%), 3 in-frame indels (3%), 3 stop-gained variants (3%), 1 frameshift variant (1%), 1 splice-site variant (1%), the remaining 22 variants (23%) being synonymous (Table 3). All 95 rare variants were identified only once in our database except 5 variants (*MAP2K1* c.315C>T, *MYBPC3* c.1957_1962delGGCCGC, *MYBPC3* c.1965A>G*, MYBPC3* c.1967C>T, *MYBPC3* c.3413G>C,), which were detected twice.

Among all variants, 43 (45%) were not previously published nor reported in online variant databases. Molecular consequences at the sequence level of novel variants are enumerated in Table 4.

As for the already reported variants (*n* = 52.55%), 6 of these were classified as pathogenic/likely pathogenic, 14 were variant of uncertain significance, and 11 were benign/likely benign according to the ClinVar archive; 8 variants had conflicting interpretations of pathogenicity (CON), either VUS + LP (2 cases) or VUS + LB/B (6 cases). For 13 rare variants, the ClinVar classification was not available. The positive tests were due to P/LP variants in the *MYBPC3* and *MYH7* genes (2 cases each), *TNNI3* and *TPM1* accounting for the remaining 2 cases (Table 5, P/LP variants represented in bold letters).

Multiple variants were detected in 27 (60%) patients, with a maximum of 11 variants in a single subject. No proband had more than one LP/P variant.

## 4. Discussion

In this study, we explored the genetic basis of a small cohort of Romanian adult index patients with HCM. The general characteristics of our study cohort were similar with data reported by Romanian Registry of Hypertrophic Cardiomyopathy [19], with an average age at enrolment falling in the fifth decade of life, and with male predominance.

In a nutshell, the main findings of our research comprised detection of 95 different rare variants in 33 genes of the 47 genes studied. *MYBPC3* and *TTN* showed the greatest sequence variation. The extensive variation of TTN could have been predicted seen the size of the protein and the numerous alternative splicing the gene undergoes to encode various isoforms. Targeted sequencing revealed a definite genetic etiology (P or LP variant) in 6 subjects (13.3%) and a possible etiology due to known variations (either VUS or CON variants favoring pathogenicity) in an additional 35.6% (*n* = 16). All P/LP variants were found in genes encoding sarcomere proteins. Almost half of the rare variants spotted were novel.

In our study, the detection rate of LP/P variants was lower than data specified by prior studies [20]. There are several valid explanations of this phenomenon. First, more stringent criteria for variant classification have been applied lately, including segregation and/or population data as recommended by ACMG [17]. Hence, irrespective of the geographic region of origin, yield of positive genetic testing progressively declined with time, from 57.7% before 2000 to 38.4% after 2010, as shown in an analysis from a large international registry [21].

The first large-scale systematic screening of genes for causal mutations for HCM revealed disease-causing variants in 63% of unrelated index cases with familial or sporadic disease. Similar detection rates (64%) were obtained by Lopes and colleagues who used high-throughput sequencing of 41 genes in 223 unrelated patients with HCM [10]. High prevalence of pathogenic mutations (67%) was also evidenced in a nationwide study on 141 Icelandic patients with clinical diagnosis of HCM [11], while in more recent studies P/LP variants were found within 21.4% to 38% of cases [12,13,22,23,24]. Secondly, increased referral for genetic testing have been prompted lately, including cases with less severe phenotypes and/or less conclusive diagnosis [22,25].

Thirdly, there is only scarce data regarding the genetic basis of HCM in Romanian population, the limited available data being related mainly to phenocopies [26,27,28,29].

Forty-five percentage of rare variants identified in our study were novel, and all (except *MYBPC3* c.1965A>G and *MYBPC3* c.1957_1962delGGCCGC) were “private”, each found only once in our cohort. Some of them might be eventually proven to be disease-causing, but definitive classification is challenging and the timeline may be indeterminate, requiring additional studies based on informative segregation analysis of comprehensive pedigrees. The proportion of novel variants in our cohort is comparable with literature data indicating a burden of 35–40% owed to newly noticed mutations, half being unique for a family [22].

As for genes harboring LP/P mutations, our data is consistent with extensive prior findings showing that the most frequent causative variants were detected in core sarcomeric genes, predominantly *MYBPC3* and *MYH7* which together explain approximately half of the cases of familial HCM [30,31,32].

Sixteen probands (35.6%) in our cohort carried a known VUS or CON variant (VUS/LP) without another likely causal variant, a higher rate than recently published by a Finnish group [12]. Five subjects (11%) harbored previously reported variants for which ClinVar classification was not available (with or without one or more novel variants), while another 5 patients had only novel variants. Altogether, these inconclusive results accounted for 68.9% of total cases, consistently with published data showing inconclusive or negative test results in 40 to 60% of screened subjects [20,33,34,35,36].

For the remaining 8 patients (17.8%) from our cohort, no variant (P/LP, VUS, CON or novel) was detected in any of the genes tested, indicating that additional studies might be needed in order to elucidate the underlying molecular substratum.

The failure to identify rare Mendelian variants in a substantial proportion of HCM patients suggests that more complex etiologies are likely to underlie this illness [37]. Recently, several hypotheses addressed this topic.

1. HCM caused by rare variants in unknown genes for HCM. In the quest to identify putative causative variants outside of recognized HCM genes, various groups used extended next-generation sequencing gene panels or even whole exome/genome sequencing (WES/WGS) as a first/second-line genetic test. In a Dutch study including 453 HCM patients, the sensitivity of genetic testing only slightly improved with the increasing number of genes sequenced, but prompted primarily the yield of class 3 variants (49%) [13]. Likewise, considerable increased detection of VUS (99%) was reported by Thomson and colleagues after examining 51 genes in 240 sarcomere gene negative HCM individuals and 6229 controls, with negligible incremental diagnostic yield [38]. In light of aforementioned findings, one can assert that expanded gene panels appear to offer limited additional sensitivity, most of genes within diagnostic tests lacking robust evidence of disease association [7,35].
2. HCM caused by rare variants in regulatory non-coding regions of already recognized causal genes. In a paper published in 2018 by Bagnall and colleagues, it has been demonstrated that variation within deep intronic regions of *MYBPC3* can explain up to 9% of gene-elusive HCM cases [39].
3. HCM caused by rare variants in mitochondrial DNA (mtDNA). Although rare or even private mtDNA mutations are frequently encountered in HCM patients [40], only rarely they are directly associated with the disease [38], more often acting as disease modifiers rather than cause [41].
4. Non-Mendelian HCM. A growing body of evidence indicates that genotype-negative HCM cases are most likely to represent non-Mendelian forms of disease, with less severe prognosis and lower risk to relatives [42]. The ability to accurately identify and characterize such candidate variants is encumbered by the necessity to perform genome-wide association studies in large cohorts assessing both variant frequency in the population and phenotypic effect size in patients [37].

In line with evidence reported by Burns and colleagues [23], no proband had multiple LP/P variants, but various combinations of LP/P and VUS or VUS/VUS with or without novel detected variants, implying that the actual incidence of multiple LP/P carriers in HCM might be lower than stated in early studies [32,43,44,45,46]. Indeed, in a study comprising 1411 unrelated index cases, after rigorous variant curation according to current guidelines, the prevalence of multiple LP/P mutations diminished substantially (from 9 to 0.4%).

### Strengths and Limitations of the Study

Our study benefits from the following strong points:

- Use of a comprehensive panel including 47 genes associated with HCM.
- Screening for the first time of a cohort of Romanian index cases.

The study is encumbered by reduced number of enrolled patients.

Future perspectives:

- Validation of the identified variants through Sanger sequencing.
- Expanding the study cohort.
- Performing segregation analyses both for known and novel variants.
- Conducting functional studies for novel detected variants.
- Checking for rare variants in the remaining genes of the TruSight Cardio Sequencing panel.

## 5. Conclusions

To our knowledge, this is the first study exploring an extensive panel of HCM-related genes in a cohort of Romanian index patients. All disease-causing variants were detected in four genes encoding sarcomere proteins. The clinical significance of most detected variants is yet to be established, additional studies based on segregation analysis being required for a definite classification.

## Figures and Tables

**Table 1:** List of the 47 genes analyzed in our study and the number of rare variants (AF < 0.001) identified per gene (core sarcomeric genes are represented in bold letters).

Gene	Chromosome	Encoding Protein	Number of Rare Variants Identified
ACTA1	1	Actin alpha skeletal muscle	1
**ACTC1**	**15**	**Actin alpha cardiac muscle 1**	**0**
ACTN2	1	Actinin alpha 2	3
ANKRD1	10	Ankyrin repeat domain-containing protein 1	2
BRAF	7	Serine/threonine-protein kinase B-raf	1
CALR3	19	Calreticulin 3	1
CASQ2	1	Calsequestrin 2	0
CAV3	3	Caveolin-3	1
COX15	10	Cytochrome c oxidase assembly protein COX15 homolog	0
CRYAB	11	Alpha-crystallin B chain	0
CSRP3	11	Cysteine and glycine-rich protein 3	1
DES	2	Desmin	4
FHL1	X	Four and a half LIM domains protein 1	0
FXN	9	Frataxin	0
GAA	17	Lysosomal alpha-glucosidase	3
GLA	X	Alpha-galactosidase A	0
JPH2	20	Junctophilin-2	2
KLF10	8	Krueppel-like factor 10	2
LAMP2	X	Lysosome-associated membrane glycoprotein 2	1
LDB3	10	LIM domain-binding protein 3	5
MAP2K1	15	Dual specificity mitogen-activated protein kinase kinase 1	1
MAP2K2	19	Dual specificity mitogen-activated protein kinase kinase 2	0
**MYBPC3**	**11**	**Myosin-binding protein C, cardiac-type**	**17**
MYH6	14	Myosin heavy chain 6	3
**MYH7**	**14**	**Myosin heavy chain 7**	**9**
**MYL2**	**12**	**Myosin regulatory light chain 2**	**1**
**MYL3**	**3**	**Myosin light chain 3**	**0**
MYLK2	20	Myosin light chain kinase 2	1
MYO6	6	Myosin-VI	1
MYOZ2	4	Myozenin-2	1
MYPN	10	Myopalladin	1
NEXN	1	Nexilin	1
PDLIM3	4	PDZ and LIM domain protein 3	1
PLN	6	Cardiac phospholamban	0
PRKAG2	7	5′-AMP-activated protein kinase subunit gamma-2	2
PTPN11	12	Tyrosine-protein phosphatase non-receptor type 11	0
RAF1	3	RAF proto-oncogene serine/threonine-protein kinase	0
SLC25A4	4	ADP/ATP translocase 1	0
SOS1	2	Son of sevenless homolog 1	2
TCAP	17	Telethonin	1
TNNC1	3	Troponin C	0
**TNNI3**	**19**	**Troponin I**	**1**
**TNNT2**	**1**	**Troponin T**	**4**
**TPM1**	**15**	**Tropomyosin alpha-1 chain**	**2**
TRIM63	1	E3 ubiquitin-protein ligase TRIM63	1
TTN	2	Titin	17
VCL	10	Vinculin	1

AF allele frequency.

**Table 2:** General and echocardiographic characteristics of HCM subjects with (G+) or without (G−) definitive genetic results.

Variable	G+ (*n* = 6)	G− (*n* = 39)	*p*
Age at inclusion, years	34 ± 10.3	53 ± 14.7	0.04
Sex: male, n (%)	6 (100%)	27 (69.2%)	0.31
Family history of HCM, n (%)	2 (33.3%)	5 (12.82%)	0.06
Family history of SCD, n (%)	4 (66.7%)	10 (25.6%)	0.065
ICD, n (%)	1 (16.7%)	6 (15.4%)	0.68
Atrial fibrillation, n (%)	5 (83.33%)	17 (43.6%)	0.35
Echocardiographic data			
Maron classification, n (%)			
1	2 (33.3%)	5 (17.9%)	0.56
2	1 (16.7%)	4 (10.3%)	
3	3 (50%)	29 (69.2%)	
4	0	1 (2.6%)	
Presence of LVOTO, n (%)	1 (16.7%)	19 (48.7%)	0.29
LV maximal wall thickness, mm	18.83 ± 7.28	20.97 ± 4.88	0.36
LV mass, g	262.4 ± 113.7	275.45 ± 96	0.53
LVEDD, mm	46.2 ± 9	39.9 ± 7.17	0.13
LVESD, mm	26 ± 7.29	24 ± 10.8	0.66
LVEDV, ml	106.85 ± 37.33	121.6 ± 44.22	0.43
LVESV, ml	50.96 ± 26.82	55.4 ± 21.3	0.64
LVEF, (%)	58.52 ± 19.9	56.6 ± 13.36	0.76
LAD, mm	39.8 ± 5.49	40.74 ± 7	0.77
LAV, ml	117.8 ± 68.18	83.19 ± 41.9	0.12

HCM hypertrophic cardiomyopathy; ICD internal cardiac defibrillator; LAD left atrium diameter; LAV left atrium volume; LV left ventricular; LVEDD left ventricular end-diastolic diameter; LVEDV left ventricular end-diastolic volume; LVEF left ventricular ejection fraction; LVESD left ventricular end-systolic diameter; LVESV left ventricular end-systolic volume; LVOTO left ventricular outflow tract obstruction; PW posterior wall; SCD sudden cardiac death.

**Table 3:** Summary of rare variants (AF < 0.001) identified in our cohort.

Consequence	Missense	Stop-Gained	In-Frame	Frameshift	Splice	Synonymous	Total
Previously reported	35	1	2	-	-	14	52
Novel	30	2	1	1	1	8	43
Total	65	3	3	1	1	22	95

AF allele frequency.

**Table 4:** Novel rare variants (AF < 0.001) detected in our cohort; variants classified by VarSome as LP/P are represented in bold letters.

Gene	HGVSc	HGVSp	Molecular Consequence	In Silico Predictions	VarSome Class	No. Cases
**ACTA1**	**c.848G>A**	**p.Ser283Asn**	**Missense variant**	**S: D** **P: N** **PP: B** **MT: DC**	**LP**	**1**
ACTN2	c.411C>A	p.Ile137=	Synonymous variant	S: T P: N PP: NA MT: DC	LB	1
ACTN2	c.973G>T	p.Asp325Tyr	Missense variant	S: D P: D PP: PrD MT: DC	VUS	1
ANKRD1	c.566C>T	p.Ala189Val	Missense variant	S: D P: D PP: PoD MT: DC	VUS	1
**CALR3**	**c.877G>T**	**p.Glu293Ter**	**Stop gained**	**S: D** **P: NA** **PP: NA** **MT: DC**	**P**	**1**
DES	c.462C>A	p.Leu154=	Synonymous variant	S: T P: N PP: NA MT: DC	LB	1
**DES**	**c.1023T>G**	**p.Thr341=**	**Synonymous variant**	**S: T** **P: N** **PP: NA** **MT: DC**	**LP**	**1**
**DES**	**c.1095C>A**	**p.Asp365Glu**	**Missense variant**	**S: T** **P: N** **PP: B** **MT: DC**	**LP**	**1**
DES	c.1104G>T	p.Ala368=	Synonymous variant	S: T P: N PP: NA MT: Pol	LB	1
GAA	c.352G>A	p.Gln118Lys	Missense variant	S: T P: N PP: B MT: Pol	VUS	1
JPH2	c.1683G>T	p.Ala561=	Synonymous variant	S: T P: N PP: NA MT: DC	LB	1
JPH2	c.1039G>T	p.Val347Phe	Missense variant	S: D P: D PP: PrD MT: DC	LB	1
KLF10	c.1060G>T	p.Ala354Ser	Missense variant	S: T P: N PP: B MT: Pol	VUS	1
LDB3	c.563G>A	p.Gly188Asp	Missense variant	S: T P: N PP: B MT: Pol	LB	1
LDB3	c.1103C>A	p.Pro368His	Missense variant	S: T P: N PP: NA MT: DC	LB	1
LDB3	c.1155C>A	p.Thr385=	Synonymous variant	S: T P: N PP: NA MT: Pol	LB	1
LDB3	c.1838C>A	p.Pro613Gln	Missense variant	S: D P: D PP: NA MT: DC	VUS	1
**MYBPC3**	**c.2813C>T**	**p.Ala938Val**	**Missense variant**	**S: D** **P: N** **PP: PrD** **MT: DC**	**LP**	**1**
MYBPC3	c.1965A>G	p.Ile655Met	Missense variant	S: T P: N PP: B MT: Pol	VUS	2
**MYBPC3**	**c.1957_1962delGGCCGC**	**p.Gly653_Arg654del**	**In-frame deletion**	**S: NA** **P: D** **PP: NA** **MT: Pol**	**LP**	**2**
MYBPC3	c.1252A>C	p.Lys418Gln	Missense variant	S: T P: N PP: B MT: DC	VUS	1
MYBPC3	c.1251C>T	p.Ala417=	Synonymous variant	S: T P: N PP: NA MT: DC	LB	1
**MYBPC3**	**c.1247_1248insCCAG**	**p.Ala417GlnfsTer29**	**Frameshift variant**	**S: NA** **P: NA** **PP: NA** **MT: DC**	**P**	**1**
MYBPC3	c.996G>T	p.Glu332Asp	Missense variant	S: T P: N PP: B MT: DC	VUS	1
MYH6	c.2571G>T	p.Glu857Asp	Missense variant	S: T P: N PP: PrD MT: DC	LB	1
MYH6	c.2346G>T	p.Arg782Ser	Missense variant	S: D P: D PP: B MT: DC	VUS	1
MYLK2	c.1431C>A	p.Ser477Arg	Missense variant	S: D P: D PP: PrD MT: DC	VUS	1
MYOZ2	c.236C>A	p.Ala79Glu	Missense variant	S: T P: N PP: PoD MT: DC	LB	1
NEXN	c.44C>A	p.Ser15Tyr	Missense variant	S: D P: N PP: PoD MT: DC	VUS	1
PRKAG2	c.1381C>T	p.Pro461Ser	Missense variant	S: D P: D PP: PrD MT: DC	VUS	1
SOS1	c.3434A>G	p.Asp1145Gly	Missense variant	S: T P: N PP: B MT: DC	VUS	1
TCAP	c.68C>A	p.Ala23Glu	Missense variant	S: D P: D PP: PoD MT: DC	VUS	1
TRIM63	c.697C>A	p.Gln233Lys	Missense variant	S: T P: N PP: B MT: Pol	LB	1
TTN	c.44530G>T	p.Ala14844Ser	Missense variant	S: D P: N PP: PrD MT: DC	VUS	1
TTN	c.30392G>T	p.Cys10131Phe	Missense variant	S: T P: D PP: B MT: DC	VUS	1
TTN	c.26928G>T	p.Leu8976=	Synonymous variant	S: T P: N PP: NA MT: DC	LB	1
TTN	c.25185G>T	p.Lys8395Asn	Missense variant	S: D P: D PP: PrD MT: DC	LB	1
**TTN**	**c.22816+1G>T**		**Splice donor variant**	**S: NA** **P: NA** **PP: NA** **MT: DC**	**P**	**1**
TTN	c.16783G>T	p.Val5595Leu	Missense variant	S: T P: N PP: B MT: Pol	LB	1
TTN	c.11927A>G	p.Lys3976Arg	Missense variant	S: T P: N PP: B MT: Pol	LB	1
**TTN**	**c.11338G>T**	**p.Glu3780Ter**	**Stop gained**	**S: NA** **P: NA** **PP: NA** **MT: DC**	**P**	**1**
TTN	c.2518G>T	p.Ala840Ser	Missense variant	S: D P: N PP: B MT: DC	VUS	1
TTN	c.49G>T	p.Val17Leu	Missense variant	S: T P: N PP: B MT: DC	VUS	1

AF allele frequency; B benign; D damaging (SIFT)/ deleterious (Provean); DC disease causing; LB likely benign; LP likely pathogenic; N neutral; NA not available; P pathogenic; PoD possibly damaging; Pol polymorphism; PrD probably damaging; T tolerated; VUS variant of uncertain significance.

**Table 5:** Previously reported rare variants (AF < 0.001) detected in our cohort; LP/P variants are represented in bold letters.

Gene	HGVSc	HGVSp	dbSNP ID	ClinVar ID	ClinVar Class	No. Cases
ACTN2	c.2445C>T	p.Ile815=	rs397516575	43929	LB	1
ANKRD1	c.197G>A	p.Arg66Gln	rs150797476	45628	LB	1
BRAF	c.95_100dupGCGCCG	p.Gly32_Ala33dup	rs397515331	41448	VUS	1
CAV3	c.39C>T	p.Ile13=	rs200562715	179005	LB	1
CSRP3	c.208G>T	p.Gly70Trp	rs777211110	520335	VUS	1
GAA	c.762G>A	p.Ser254=	rs533960093	509666	LB	1
GAA	c.899C>A	p.Ala300Glu	rs1032949450	NA	NA	1
KLF10	c.973G>A	p.Val325Ile	rs760040811	NA	NA	1
LAMP2	c.37G>T	p.Gly13Trp	rs12853266	NA	NA	1
LDB3	c.610G>A	p.Ala204Thr	rs774976112	626705	CON (LB/VUS)	1
MAP2K1	c.315C>T	p.Pro105=	rs144166521	44589	B	2
MYBPC3	c.3413G>C	p.Arg1138Pro	rs187705120	42712	VUS	2
**MYBPC3**	**c.3294G>A**	**p.Trp1098Ter**	**rs767039057**	**520341**	**P**	**1**
MYBPC3	c.3262C>G	p.Pro1088Ala	rs1263358939	NA	NA	1
MYBPC3	c.2882C>T	p.Pro961Leu	rs373056282	42665	VUS	1
MYBPC3	c.2441_2443delAGA *	p.Lys814del *	rs727504288	177700	CON (VUS/LP)	1
MYBPC3	c.1967C>T	p.Pro656Leu	rs927421140	NA	NA	2
MYBPC3	c.1316G>A	p.Gly439Asp	rs763045718	628463	VUS	1
MYBPC3	c.1127G>A	p.Ser376Asn	rs1595846858	NA	NA	1
**MYBPC3**	**c.772G>A**	**p.Glu258Lys**	**rs397516074**	**42792**	**P**	**1**
MYBPC3	c.152C>T	p.Ala51Val	rs746738538	NA	NA	1
MYH6	c.2710G>T	p.Glu904Ter	rs759822161	NA	NA	1
MYH7	c.5736C>T	p.Ile1912=	rs200728597	43086	B	1
MYH7	c.5203T>A	p.Ser1735Thr	rs144066768	181272	VUS	1
MYH7	c.4377G>T	p.Lys1459Asn	rs201307101	43012	LB	1
MYH7	c.4348G>A	p.Asp1450Asn	rs397516211	43009	VUS	1
MYH7	c.4212G>T	p.Val1404=	rs397516205	43000	LB	1
**MYH7**	**c.2389G>A**	**p.Ala797Thr**	**rs3218716**	**42901**	**LP/P**	**1**
MYH7	c.1755C>T	p.Ile585=	rs201860580	194465	CON (LB/VUS)	1
MYH7	c.1108G>A	p.Glu370Lys	NU	858379	VUS	1
**MYH7**	**c.715G>A**	**p.Asp239Asn**	**rs397516264**	**43100**	**LP/P**	**1**
MYL2	c.374C>T	p.Thr125Met	rs375667565	43473	VUS	1
MYO6	c.2322T>C	p.Pro774=	rs947653207	NA	NA	1
MYPN	c.1012C>T	p.Arg338Cys	rs140037748	201882	VUS	1
PDLIM3	c.334G>A	p.Gly112Arg	rs777447396	967683	VUS	1
PRKAG2	c.147C>T	p.Asp49=	rs761196275	696154	LB	1
SOS1	c.661C>G	p.Leu221Val	rs1007628403	NA	NA	1
**TNNI3**	**c.557G>A**	**p.Arg186Gln**	**rs397516357**	**43395**	**LP/P**	**1**
TNNT2	c.863G>A	p.Arg288His	rs397516484	43674	VUS	1
TNNT2	c.774C>T	p.Phe258=	rs397516481	43668	LB	1
TNNT2	c.430C>T	p.Arg144Trp	rs45525839	127070	VUS	1
TNNT2	c.341C>T	p.Ala114Val	rs727504245	177633	CON (VUS/LP)	1
**TPM1**	**c.574G>A**	**p.Glu192Lys**	**rs199476315**	**31882**	**P**	**1**
TPM1	c.835C>T	p.Leu279=	rs374434837	378751	LB	1
TTN	c.40423A>G	p.Lys13475Glu	rs775980062	NA	NA	1
TTN	c.32736G>A	p.Pro10912=	rs368838709	NA	NA	1
TTN	c.29079G>A	p.Ala9693=	rs372997298	137775	CON (B/LB/VUS)	1
TTN	c.22386T>A	p.Asp7462Glu	rs183482849	46699	CON (B/VUS)	1
TTN	c.20395C>T	p.Arg6799Trp	rs751534449	809053	VUS	1
TTN	c.15856G>A	p.Gly5286Ser	rs1409273228	NA	NA	1
TTN	c.11959A>G	p.Ile3987Val	rs551387805	264496	CON (LB/VUS)	1
VCL	c.3186G>A	p.Gln1062=	rs761534024	300798	CON (LB/VUS)	1

AF allele frequency; B benign; CON variant with conflicting interpretations of pathogenicity; LB likely benign; LP likely pathogenic; NA data not available; P pathogenic; VUS variant of uncertain significance. * GenBank accession number MH595891, variant previously published by our group in [14].

## Abbreviations

ACMG	American College of Medical Genetics and Genomics
ACTA1	Actin alpha skeletal muscle
ACTC1	Actin alpha cardiac muscle 1
ACTN2	Actinin alpha 2
ANKRD1	Ankyrin repeat domain-containing protein 1
AMP	Association for Molecular Pathology
B	benign
*BRAF*	Serine/threonine-protein kinase B-raf
BWA-MEM	Burrows-Wheeler Aligner-Maximal Exact Match
CALR3	Calreticulin 3
CASQ2	Calsequestrin 2
CAV3	Caveolin-3
COX15	Cytochrome c oxidase assembly protein COX15 homolog
CRYAB	Alpha-crystallin B chain
CSRP3	Cysteine and glycine-rich protein 3
DES	Desmin
DNA	deoxyribonucleic acid
ESC	European Society of Cardiology
FHL1	Four and a half LIM domains protein 1
FXN	Frataxin
GAA	Lysosomal alpha-glucosidase
GATK	Genome Analysis Toolkit
GLA	Alpha-galactosidase A
HCM	hypertrophic cardiomyopathy
HGMD	Human Gene Mutation Database
JPH2	Junctophilin-2
KLF10	Krueppel-like factor 10
LAMP2	Lysosome-associated membrane glycoprotein 2
LB	likely benign
LDB3	LIM domain-binding protein 3
LP	likely pathogenic
LV	left ventricle
LVH	left ventricular hypertrophy
MAP2K1	Dual specificity mitogen-activated protein kinase kinase 1
MAP2K2	Dual specificity mitogen-activated protein kinase kinase 2
mtDNA	mitochondrial DNA
MYBPC3	cardiac myosin binding protein C
MYH6	Myosin heavy chain 6
MYH7	β-myosin heavy chain
MYL2	Myosin regulatory light chain 2
MYL3	Myosin light chain 3
MYLK2	Myosin light chain kinase 2
MYO6	Myosin-VI
MYOZ2	Myozenin-2
MYPN	Myopalladin
NEXN	Nexilin
NGS	next generation sequencing
P	pathogenic
PDLIM3	PDZ and LIM domain protein 3
PLN	Cardiac phospholamban
PRKAG2	5′-AMP-activated protein kinase subunit gamma-2
PTPN11	Tyrosine-protein phosphatase non-receptor type 11
RAF1	RAF proto-oncogene serine/threonine-protein kinase
SLC25A4	ADP/ATP translocase 1
SOS1	Son of sevenless homolog 1
TCAP	Telethonin
TNNC	Troponin C
TNNI3	Troponin I
TNNT2	Troponin T
TPM1	Tropomyosin alpha-1 chain
TRIM63	E3 ubiquitin-protein ligase TRIM63
TTN	Titin
VCF	variant call format
VCL	vinculin
VUS	variant of uncertain significance
